# Modern Dental Materia Medica

**Published:** 1912-02-15

**Authors:** 


MODERN DENTAL MATERIA MEDICA. The third edition of Buckley's Materia Medica has added another valuable manual to the good text books for students and practitioners. This work, as its predecessors, appeals more to students than practitioners, as it treats many topics with such brevity as would hardly meet the demands of the practitioner seeking a complete statement such as is usually found in a scientific treatise on any subject. Many of the drugs mentioned are so briefly discussed that no more than an epitome is made; it is true that some are so infrequently used, or are of such small value as therapeutic remedies that such treatment is sufficient. But there are some remedies that when one wants to utilize them he desires to know all that can be made known by any one. Such for illustration are the General Anesthetics, Ether, Chloroform, Somnoform and Nitrous Oxide. There is no work on dental materia medica today which gives a good clear statement of what is known about these drugs and their therapeutic uses. Dentists are timid about using them because they do not know them. We do not therefore wholly sympathize with the authors idea that he should keep down the size of the book. We should much prefer that in future editions he amplify rather than condense his consideration of the more frequently used systemic as well as the invaluable local remedies. Dr. Buckley is to be congratulated on the general arrangement of the work and the direct manner he has dealt with the large number of new and old dental remedies. The publishers have done their work exceptionally well and deserve our commendation and thanks.

